# Propofol is Effective to Depress Fentanyl-Induced Cough during Induction of Anesthesia

**DOI:** 10.5812/aapm.8383

**Published:** 2013-03-26

**Authors:** Abbas Sedighinejad, Bahram Naderi Nabi, Mohammad Haghighi, Vali Imantalab, Sodabe Hadadi, Reza Erfani Sayar, Ahmadreza Mirblook

**Affiliations:** 1 Anesthesiology Department, Guilan University of Medical Sciences, Rasht, Iran; 2 Orthopaedy Department, Guilan University of Medical Sciences, Rasht, Iran

**Keywords:** Fentanyl, Cough, Propofol, Anesthesia

## Abstract

**Background:**

Various attempts have been made to reduce the incidence of fentanyl-induced cough during anesthesia induction. We hypothesized that an appropriate dose of propofol might suppress fentanyl-induced cough.

**Objectives:**

A study had been designed to observe the effects of propofol on a fentanyl-induced cough during anesthesia induction.

**Patients and Methods:**

We performed a randomized, double-blind study to evaluate the effect of the pre-emptive use of minimal dose intravenous propofol (20 mg) on the incidence of cough caused by a larger bolus of intravenous fentanyl. Group 1 patients were given fentanyl at a dosage of 4 µg/kg. Group 2 received 4µg/kg fentanyl and 20 mg propofol. The two groups were evaluated in 0, 5 and 10 second intervals following the injection of fentanyl.

**Results:**

Mean age, weight, and, height was 35 ± 10.45, 67.99 ± 10.92, and 165.33 ± 31.84 respectively. The incidence of fentanyl induced cough was 29 (74.4%) in placebo group compared with 10 (25.6%) in the propofol group. There was a significant difference in the incidence and severity of cough between group 1 and 2 (P < 0.0001). This study also showed that propofol could decrease cough incidence in patients who smoke.

**Conclusions:**

Priming dose of propofol (20mg) one minute prior to fentanyl injection was effective in suppressing a fentanyl-induced cough.

## 1. Background

Fentanyl, a synthetic opioid, is widely used for general anesthesia by anesthesiologists in the operating room however sometimes subjects develop a cough following a round of injections (1, 2). In Bohrer’s report, up to 46% of patients had reported a fentanyl-induced cough following they were delivered 7 μg/kg of fentanyl through a central venous catheter (3). Fentanyl-induced cough is not always benign and brief; it is undesirable in patients with some underlying diseases and/or conditions, such as cerebral aneurysm, head trauma, open eye injury, dissecting aorta, pneumothorax, and hypersensitive airway disease (3, 4). Former reports have demonstrated that a fentanyl-induced cough can be reduced with pretreatment of certain drugs (4-9). Strategies to decrease the occurrence of a reflex cough following an intravenous bolus of fentanyl include techniques to prolong injection time or the use of terbutaline, clonidine, dexamethasone and lidocaine (1, 4, 7, 8, 10, 11) however, these approaches are not uniformly effective. All of these medications have bronchorelaxant effects on the airway's smooth muscle (4, 5, 12). Propofol may also induce, bronchodilation (5, 13-15), therefore we hypothesized that an appropriate dose of propofol might suppress a fentanyl-induced cough.

## 2. Objectives

We designed a randomized double blind controlled study to observe the effects of propofol on fentanyl-induced cough during anesthesia induction.

## 3. Patients and Methods

### 3.1. Patient Population

The Ethics Committee of Affiliated Poursina Hospital of Gillan Medical University approved the protocol of the present study from 2011 to 2012, and informed written consent forms were obtained from all participants. The study population consisted of 110 patients of both genders, aged 25 to 60 years, with American Society of Anesthesiologists physical status I/II, scheduled to undergo elective orthopedic surgery under general anesthesia were enrolled and randomly assigned to two groups (55 patients each), using computer-generated random numbers in this study. The sample size was calculated by based on existing references value (4, 9, 14-16). Exclusion criteria included: body-weight exceeding 20% of ideal body-weight (on the basis of body mass index recommended); impaired kidney or liver function; presence of a gastric tube; or a history of asthma, chronic cough, upper respiratory tract infection in the previous 4 weeks, or treated with angiotensin-converting enzyme inhibitors, bronchodilators, or steroids in the former four weeks prior to study. We didn't exclude the smoker in order to evaluate if this dose can suppress this phenomenon in smokers.

### 3.2. Anesthesia Induction and Data Collection

Following the patient’s arrival at the operating theater, venous access was established on the nondominant hand with a 22-G intravenous cannula and connected to a T-connector for drug administration. Monitoring included electrocardiography (ECG), non-invasive blood pressure (NIBP) and pulse oxygen saturation (SpO_2_). Supplemental oxygen therapy was given by facemask (40% O_2_ 31/min) when required to maintain saturation above 95% throughout the duration of the study. Artificial oxygen supply was given immediately if SpO_2_ levels fell below 95%. All subjects received 5ml/kg normal saline prior any drug injection and hemodynamic included: systolic and diastolic blood, pressure, Spao_2_ (pulse oximetry) and heart rate was checked every five minutes. Group 1 received 4µg/kg fentanyl (prepared by Fentanyl–hamlen Pharmaceutical Co. GERMANY) and a placebo, whereas in Group 2, the patients received 20 mg Propofol (Pofol 1%, Dangkook Pharm. Co. Ltd., Korea) followed by 4µg/kg fentanyl after one minute. We decided to administer the minimal propofol dose 1 minute before the larger bolus dose of fentanyl, to ensure that the minimal dose had completed one arm-brain circulation time. The speed of fentanyl injection was about 30 seconds and another anesthesiologist who was blind to the pre-treatment, recorded the onset time (the time of the first episode of cough) as well as the severity of cough for 0, 5 and 10 seconds after fentanyl administration. Any episode of cough was classified as coughing. Severity of coughing was graded as mild [1–2], moderate [3–5] and severe [> 5] based on the number of coughs within one minute after fentanyl injection (4). Subsequently, induction of general anesthesia was commenced with propofol 1.5–2.5 mg/kg and cisatracurium 0. 2 mg/kg and the continuous infusion dose of propofol was 5 mg kg−1 h−1. The patients were manually ventilated for 3 min before tracheal intubation was started by a practiced anesthesiologist. All patients were ventilated mechanically with a tidal volume of 8 ml/kg, at a respiratory rate of 12 breaths/min. Data are expressed as mean ± standard deviation, number, proportion or percentage. Statistical analysis was performed by SPSS (Statistical Product for Social Sciences) software 17.0. The frequencies and severity of cough in patients as well as patients from two ASA classes were compared using Chi-square or Fisher’s exact test. The pair t-test was used to compare the age, weight and height among the two groups. P < 0.05 was considered to be statistically significant.

## 4. Results

Overall, we studied 114 patients, who were divided into two groups (Propofol and placebo groups). There was not any significant statistically difference in demographic data includes: age, sex, weight and height, a between the two groups (Table 1). There was a significant statistical difference in cough incidence between two groups. (Propofol group 25.6% versus placebo group 74.4%, respectively) (P = 0.0001) (Table 2). The cough number following Fentanyl administration was statistically significant between groups (P = 0.0001). The incidence of 1-2 coughs in the Propofol group was 40.9% versus the placebo group that was 59% and 3-5 coughs was 8.3% in the Propofol group versus 91.7% in the placebo group. Remarkably, the Propofol group didn’t cough more than five times. The cough interval time (the time to cough production following the injection of Fentanyl in seconds) was significantly different between groups (P = 0.0001) (Table 2). There were no coughs prior 5 seconds following injected of Fentanyl in the Propofol group and there were 8 cases with cough in the placebo group. There was 10.5 % and 89.5% in the 6-10 seconds interval in the Propofol group and the placebo group, respectively, and following 11 seconds interval, it was 66% in the Propofol group and 33.3 % in the placebo group. It showed that cough interval time was longer in the propofol group than in the placebo group. In the smoker patients, there was no cough in 72.2 % and 27.8 % in the propofol group and placebo group, respectively. There was significant difference between the two groups (P = 0.009) (Table 3). There was also no significant difference in interval time between two groups in smoker patients (P = 0.357).

**Table 1:** Demographical Data of Patients

Variables	Placebo	Propofol	P value
**Women, No. (%)**	18 (42.9)	24 (57)	0.333 a
**Men, No. (%)**	38 (53)	33 (46.5)	0.333 a
**Age, y, Mean ± SD**	34.46 ± 9.87	35.36 ± 10.45	0.125 a
**Weight, kg, Mean ± SD**	67.75 ± 8.75	68.09 ± 10.18	0.092 a
**Height, cm, Mean ± SD**	166.45 ± 6.8	167.3 ± 7.3	0.098 a

^a^Not significant

**Table 2:** The Comparison of Cough Characteristics in two Groups (placebo and drug)

Variables	Propofol	Placebo	P value
**Cough time after Fentanyl, sec, Mean ± SD**	12.09 ± 5.31	8.37 ± 3.4	0.013
**Cough + a, No. (%)**	10 (25.6)	29 (74.4)	0.00001
**Cough, Number, No. (%)**			
No	47 (63.5)	27 (36.5)	0.00001
1-2	9 (40.9)	13 (59)	0.00001
3-5	1 (8.3)	11 (91.7)	0.00001
> 5	0	5 (100)	0.00001
**Cough interval time, sec b, No. (%)**			
< 5	0	8 (100)	0.00001
6-10	2 (10.5)	17 (89.5)	0.013
> 11	8 (66.7)	4 (33.3)	0.00001

^a^The overall cough occurrence

^b^The time to cough production following Fentanyl till drug administration

**Table 3:** The Comparison of Cough Characteristics in Smoker Groups (Placebo and Drug)

Variable	Placebo	Signal	P value
**Smoking, Use +, No. (%) a**	57 (50.4)	56 (49.6)	0.831 d
**Smoking, Interval time b**	12	8.2 ± 3.73 c	0.357 d
**Smoking, No. (%)**			
No cough	13 (72.2)	5 (27.8)	0.009
1-2 cough	1 (25)	3 (75)	0.009
3-5 cough	0	5 (100)	0.009
> 5 cough	0	2 (100)	0.009
**Cough, Smoke +, No. (%)**	1 (9.1)	10 (90.9)	0.002

^a^Cigarette smoking

^b^The time to cough production after Fentanyl till drug administration

^c^Value are expressed as the mean ± standard deviation or number of case. No significant differences were found between groups

^d^Not significant

## 5. Discussion

The major finding of the present study was, the pretreatment with 20 mg of propofol might reduce fentanyl-induced cough from 51.8% to 17.5%, which was consistent with our hypothesis. The incidence of fentanyl-induced cough varies doses over wide range of 2.7%-65% and primarily depends on the fentanyl injected, the rates of injection, and the routes of injection (1, 3, 10). Lin's and his team have revealed 65% incidence of cough in patients with intravenous administration of fentanyl 2.5 µg/kg within two seconds (10, 11). Phua et al. have demonstrated that the injection of fentanyl 1.5µg/kg via a peripheral venous line elicits cough in 28% of the patients (3, 12, 17). In a cohort study of 1311 adult patients, these authors reported that increasing age, cigarette smoking, prior epidural injection of lidocaine or a priming dose of vecuronium were associated with a decreased risk of fentanyl-induced cough (4, 15, 18). Lin et al. found that only light smoking, not heavy smoking could decrease the frequency of fentanyl-induced cough, but the association between age and incidence of cough was not observed in their study (7, 11, 19). In Tang and his colleagues' study, the incidence of cough was 80%. The exact reasons for this higher incidence of cough remained unclear. However they attribute that higher incidence to the faster bolus speed of fentanyl (bolus time: 1.5 ± 0.3 seconds) and the possible bias due to the small sample size included in their study (15). The incidence of fentanyl induced cough production has not yet been well understood (1, 6, 15, 19). Various theories about fentanyl-induced cough have been recommended. Fentanyl inhibits central sympathetic out flow, causing vagal predominance, leading to cough and bronchoconstriction and bronchospasm (8, 15, 16). It can also elicit cough by stimulating irritant receptors in tracheal smooth muscles (4). Selective β2-adrenergic bronchodilators (terbutaline and salbutamol) or N-Methyl-D-Aspartat antagonists (ketamine) have been reported to reduce the incidence of fentanyl-induced cough (4, 12, 20). Propofol possessed a bronchodilation effect (13). Conti and colleagues have concluded that propofol inhibits bronchoconstriction and decreases the risk of bronchospasm during anesthesia induction (12, 18). Pizov and co-workers have shown that the incidence of wheezing was significantly reduced in asthmatic patients receiving a propofol-based induction of anesthesia compared to a barbiturate-based induction (4, 9, 16). Additionally, propofol has a significant sedative effect that may also reduce the incidence of cough (5, 13). Tang suggested propofol is effective to suppress fentanyl-induced cough in a dose-dependent manner (15). Propofol is used as a premedicant in doses of 20 mg because of its sedative and anesthetic-sparing effects, as well as attenuating airway or circulatory reflexes during anesthesia. The strength of the present study refers to the important point that we chose the dose of 20 mg to achieve satisfactory effects, and maintain hemodynamic stability. There are two limitations in our study; one of them is the fact that a dose-response experiment was not performed to determine the optimal dose of propofol that produces the maximum depression of cough without causing side effect. Another limitation that we discovered is a decreasing of cough based on smokers that was very dominant but this case was not matched by the age. Further studies are required to determine the ideal dose of propofol that will suppress cough without causing side effects and evaluate all contributing factors to decrease bias of the study. In conclusion, before giving propofol a few minutes prior to administrating fentanyl would be effective in suppressing a fentanyl-induced cough. Considering that higher dosage of propofol may be associated with a higher incidence of cardiovascular events, we recommend using 20 mg of propofol one minute before injection of fentanyl to suppress the cough during induction of anesthesia with fentanyl.
